# Supplementary material for: Oncogenic KRAS G12C: Kinetic and redox characterization of covalent inhibition
Source: J Biol Chem. 2022 Jun 24;298(8):102186. doi: 10.1016/j.jbc.2022.102186 (PMC9352912; doi:10.1016/j.jbc.2022.102186)
Supplement: Supplemental figures [file mmc1.pdf]

Oncogenic KRAS G12C: Kinetic and Redox Characterization of Covalent Inhibition

Minh V. Huynh, Derek Parsonage, Tom E. Forshaw, Venkat R. Chirasani, G. Aaron Hobbs, Hanzhi Wu, Jingyun Lee, Cristina M. Furdui, Leslie B. Poole*, Sharon L. Campbell*

Supporting Information

List of Material Included

<u>Figures</u>

Figure S1. Y137W mutation does not perturb the structural dynamics of KRAS^{G12C}.

Figure S2. Stopped-flow kinetic studies of KRAS^{CCLW} and ARS-853 at 5 °C.

Figure S3. Additional comparison of stopped-flow kinetics of KRAS^{CCLW} with ARS-853 vs. AMG 510.

Figure S4. Comparison of stopped-flow kinetics data for KRAS^{CCLW} with ARS-853 with MHT buffer versus the buffer from Hansen et al., 2018.

Figure S5. KRAS^{G12C} is rapidly oxidized after 10 min treatment with H_2O_2.

Figure S6. Glutathionylated KRAS^{G12C} and KRAS^{G12C} thiolate show altered switch dynamics.

Figure S7. Replicate simulations show similar structural ensembles for KRAS^{G12D} and KRAS^{G12C} ($-SO_2^-$).

<u>Tables</u>

Table S1. Summary of k_{inact}/K_i values obtained for inhibitors at 20 °C and 3 pH values.

Table S2. Kinetic parameters at 20 °C and pH 7.5 of KRAS^{CCLW} with ARS-853 compared with previously reported results (1).

Table S3. Binding affinity data for KRAS mutants as shown in Figure 6D.

Table S4. Thermal melt data for KRAS mutants as shown in Figure 6E.

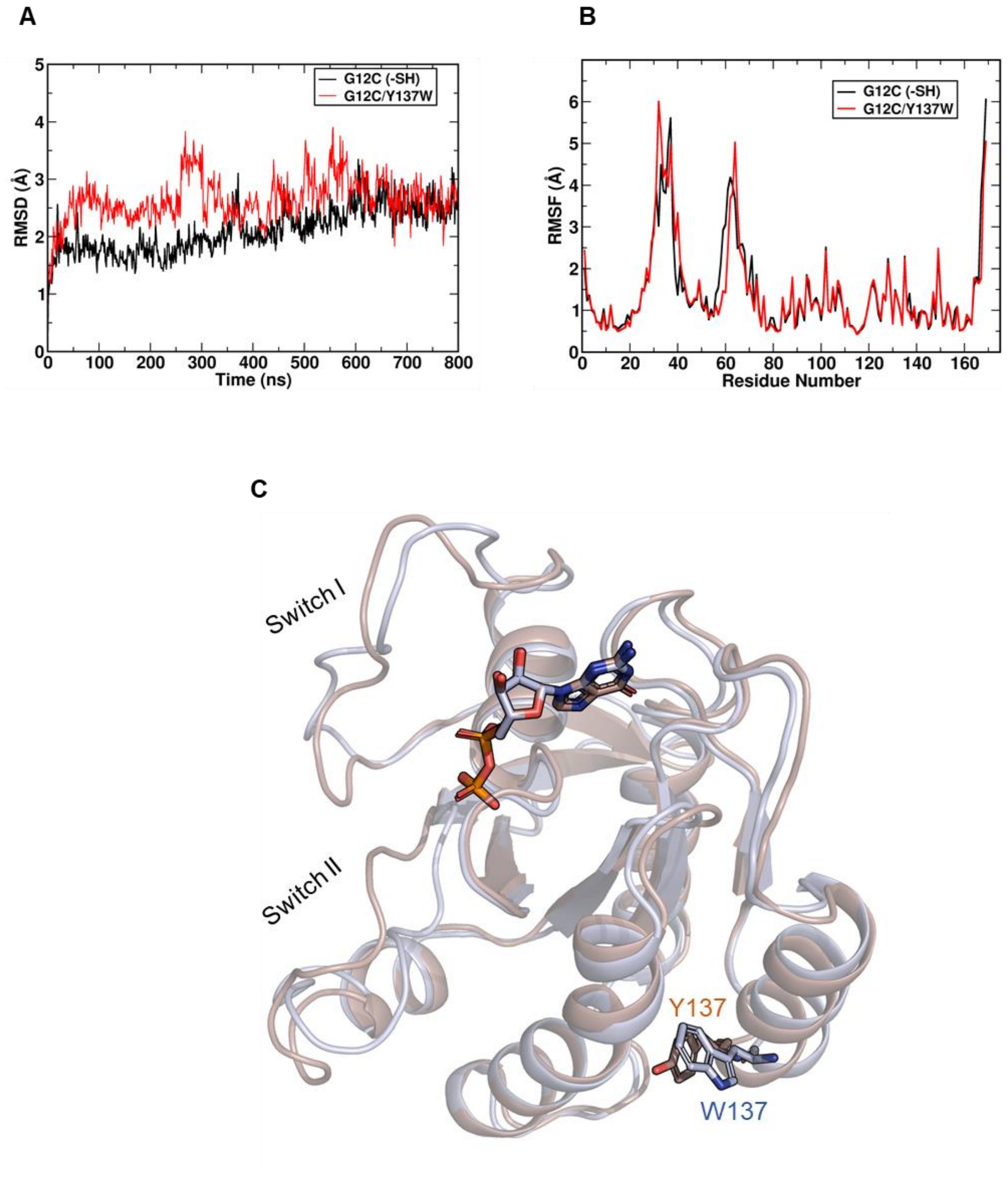

Figure S1. The KRAS$^{G12C/Y137W}$ variant shows similar structure and dynamics as KRAS^{G12C}. (**A** and **B**) RMSD and RMSF plots obtained from MD trajectories of KRAS^{G12C} and KRAS$^{G12C/Y137W}$ indicate that the Y137W substitution does not significantly alter KRAS^{G12C} structure and dynamics. **(C)** Overlay of highly populated structural ensembles of KRAS^{G12C} (brown) and KRAS$^{G12C+Y137W}$ (light blue) extracted from respective MD trajectories. Protein is shown in ribbon and GDP is shown in stick representation.

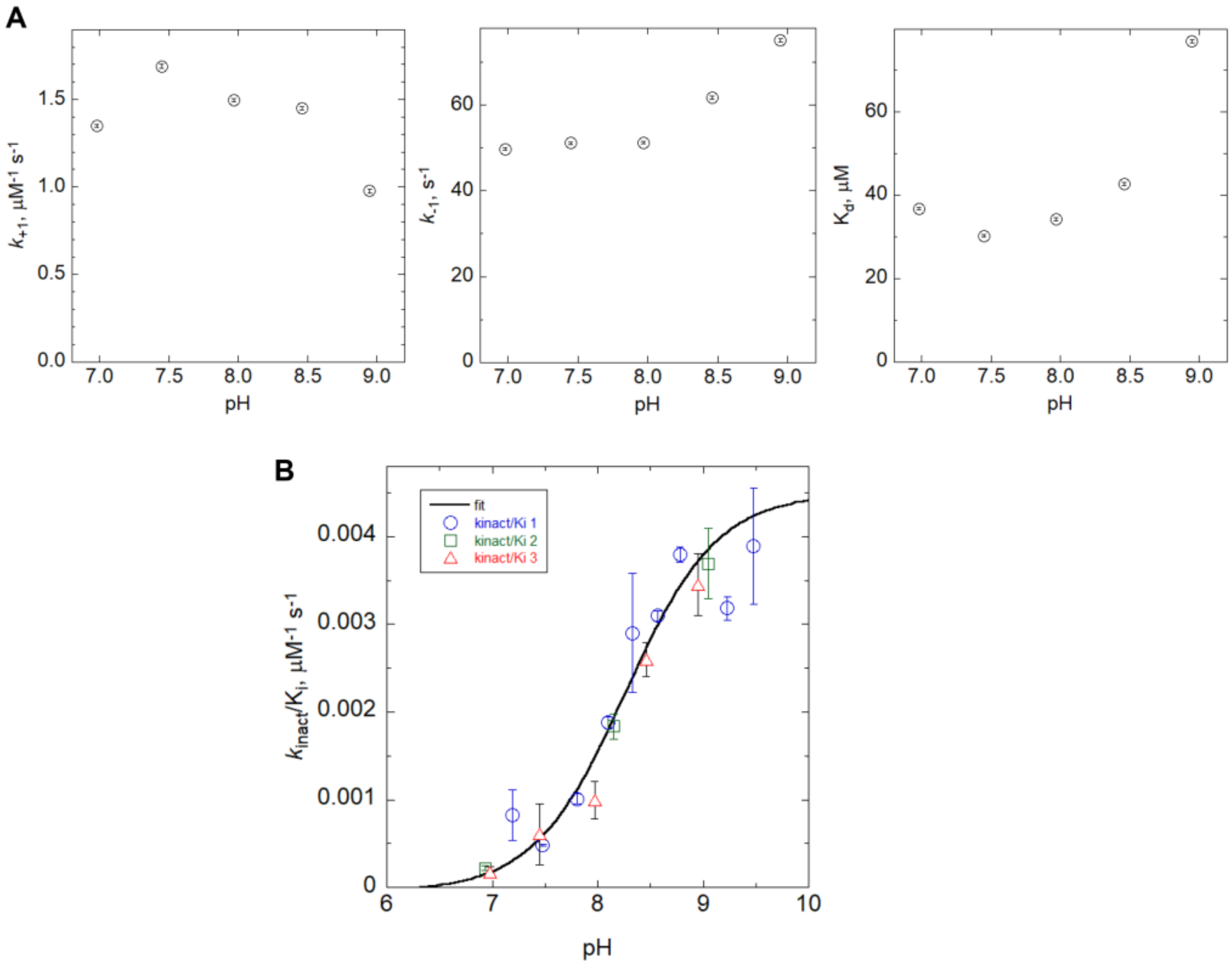

Figure S2. Stopped-flow kinetic studies of KRAS^{CCLW} and ARS-853 at 5 °C. (**A**) Kinetic rate constants and K_d for the fast step of KRAS^{CCLW} and ARS-853 show little pH sensitivity at 5 °C. (**B**) Second order rate constants (k_{inact}/K_i) for the slow step of KRAS^{CCLW} and ARS-853 show similar pH sensitivity at 5 °C as compared to 20 °C (Fig. 3E). Three colors and marker shapes represent three independent trials, all used in the final fit. Error bars, mean ± **s.e.m**.

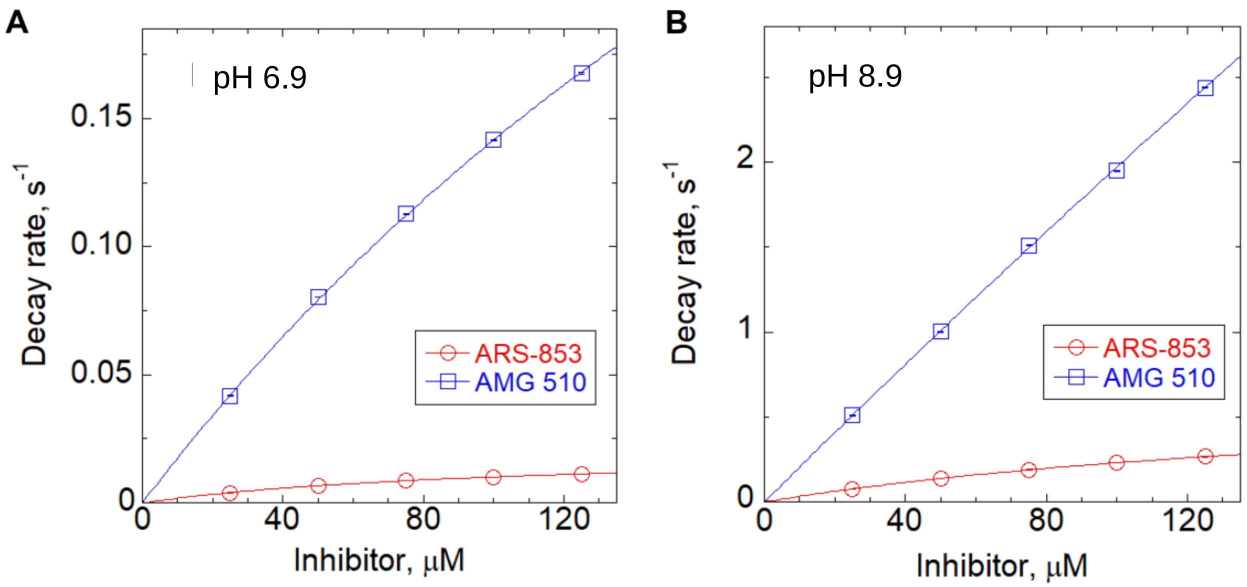

Figure S3. Additional comparison of stopped-flow kinetics of KRAS^{CCLW} with ARS-853 vs. AMG 510. (A and B) Plots of the pseudo first order rate constants versus inhibitor concentrations of KRAS^{CCLW} with AMG 510 versus ARS-853 at pH 6.9 (**A**) and pH 8.7 (**B**) at 20°C, showing fits to the "Ksp" treatment of the hyperbolic Michaelis-Menten equation as described in methods (poorly determined K_i values are similar to or higher than the highest inhibitor concentrations used). Reactions were monitored in a MES-HEPES-Tris buffer.

Table S1. Summary of k_{inact}/K_i values obtained for inhibitors at 20 °C and 3 pH values.

	pH 6.9		pH 8.6 (in Fig. 4)		pH 8.7	
	AMG 510	ARS-853	AMG 510	ARS-853	AMG 510	ARS-853
k_{inact}/K_i (M^{-1} s^{-1})	1790 (± 10)	200 (± 5)	14000 (± 100)	2770 (± 10)	20600 (± 100)	3450 (± 10)
k_{inact}/K_i (AMG vs. ARS)	9.0-fold higher		5.0-fold higher		6.0-fold higher	

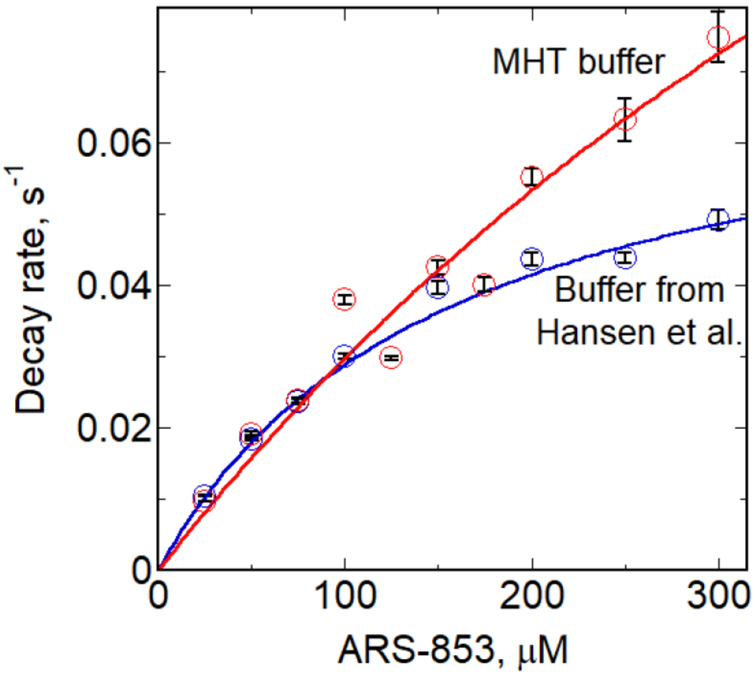

Figure S4. Comparison of stopped-flow kinetics data for KRAS^{CCLW} with ARS-853 with MHT buffer versus the buffer from Hansen et al., 2018 (1). Plots of the pseudo first order rate constants versus inhibitor concentrations of KRAS^{CCLW} with ARS-853 in a MES-HEPES-Tris (MHT) buffer (red), or the buffer composition used by Hansen et al. containing DTT (blue) at 20°C.

Table S2. Kinetic parameters at 20 °C and pH 7.5 of KRAS^{CCLW} with ARS-853 compared with previously reported results (1).

Buffer:	MHT buffer[a]	Hansen et al. buffer[b]	Literature data[c]
K_i (μM)	>500	142 ± 19	200 ± 90
k_{inact} (s^{-1})	>0.15	0.072 ± 0.004	0.05 ± 0.023
k_{inact}/K_i ($M^{-1}\ s^{-1}$)	336 ± 45	510 ± 75	250 ± 20

[a]Data were fit using the “Ksp” approach as described in Methods.
[b]Data were fit to equation 1 (in the main text).
[c]Data reported by Hansen et al. (1).

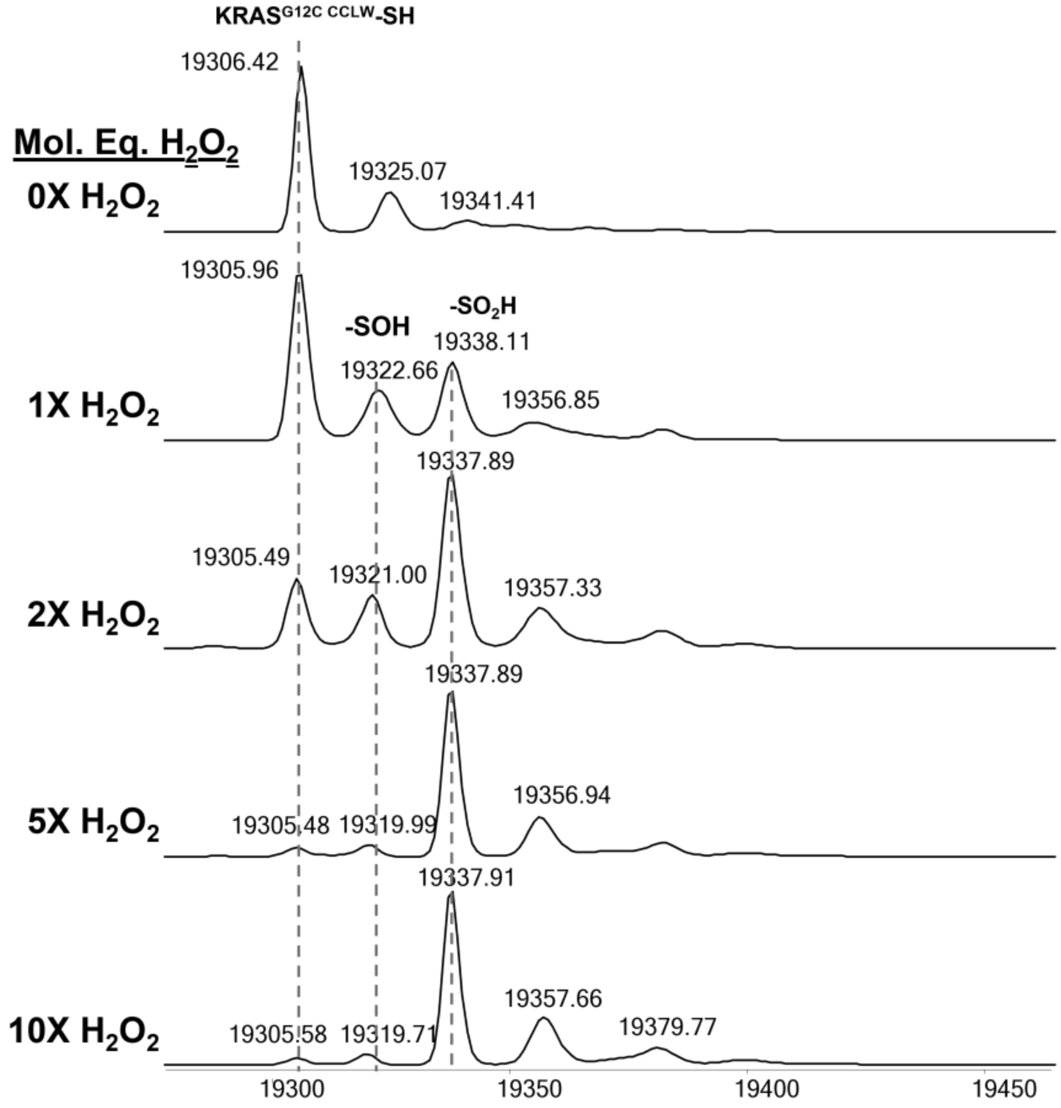

Figure S5. KRAS^{G12C} is rapidly oxidized after 10 min treatment with H_2O_2. Increasing molar equivalents of H_2O_2 show increased formation of sulfenic (-SOH) and sulfinic acid (-SO_2H) modifications.

Table S3. Binding affinity data for KRAS mutants as shown in Figure 6D.[a]

	K_d (nM)
$KRAS^{C118S}$	58 ± 6
$KRAS^{G12C/C118S}$	54 ± 15
$KRAS^{G12C/C118S}$ + GSSG	183 ± 28
$KRAS^{G12D/C118S}$	149 ± 65
$KRAS^{G12S/C118S}$	60 ± 14

[a] Calculated binding affinities of wild-type and mutant KRAS proteins in complex to BRAF-RBD as determined by inhibition of nucleotide dissociation. Data shown are averaged from three or more independent experiments.

Table S4. Thermal melt data for KRAS mutants as shown in Figure 6E.[a]

	T_m (°C)
$KRAS^{C118S}$	64.5 ± 1.5
$KRAS^{G12C/C118S}$	67.1 ± 1.1
$KRAS^{G12C/C118S}$ + CysNO	67.8 ± 2.5
$KRAS^{G12C/C118S}$ + GSSG	67.0 ± 2.8

[a] Calculated circular dichroism melting temperatures of KRAS proteins. Data shown are averaged from three or more independent experiments.

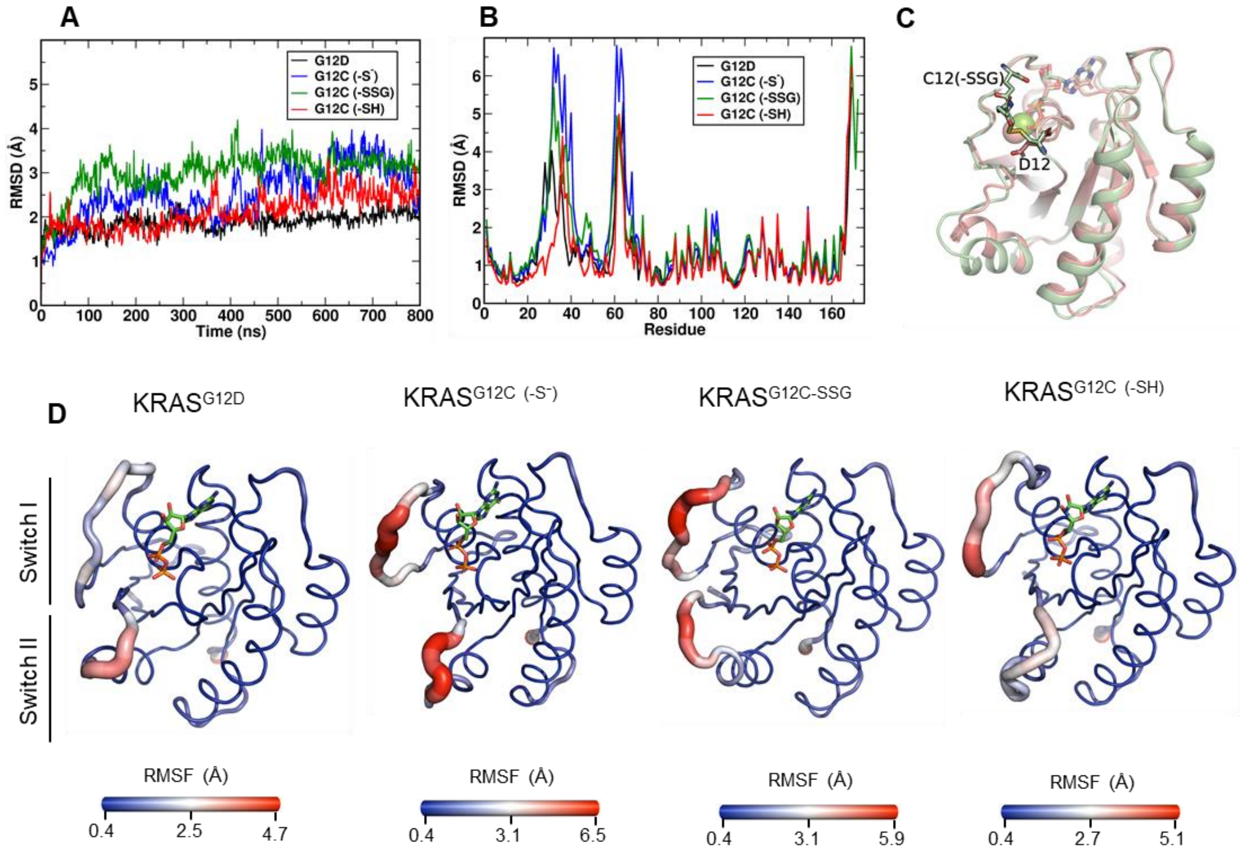

Figure S6. Glutathionylated KRAS^{G12C} and KRAS^{G12C} thiolate show altered switch dynamics. (**A** and **B**) Molecular dynamics (MD) trajectories of GDP-bound KRAS^{G12D} as well as KRAS^{G12C} in the protonated (-SH), thiolate (-S$^-$), and glutathionylated state (-SSG). **(C)** Ribbon diagram overlay of modeled KRAS^{G12C} (-SSG) (salmon) and KRAS^{G12D} (green). G12C-SSG and G12D side-chains are represented as sticks. **(D)** Sausage representation of KRAS^{G12D}, KRAS^{G12C} (-S$^-$), KRAS^{G12C} (-SSG), and KRAS^{G12C} structures extracted from respective MD trajectories. Glutathionylation of the KRAS^{G12C} thiol and deprotonatation of KRAS^{G12C} (thiolate) increase fluctuations in the KRAS Switch regions as compared to reduced KRAS^{G12C} (-SH) and KRAS^{G12D}.

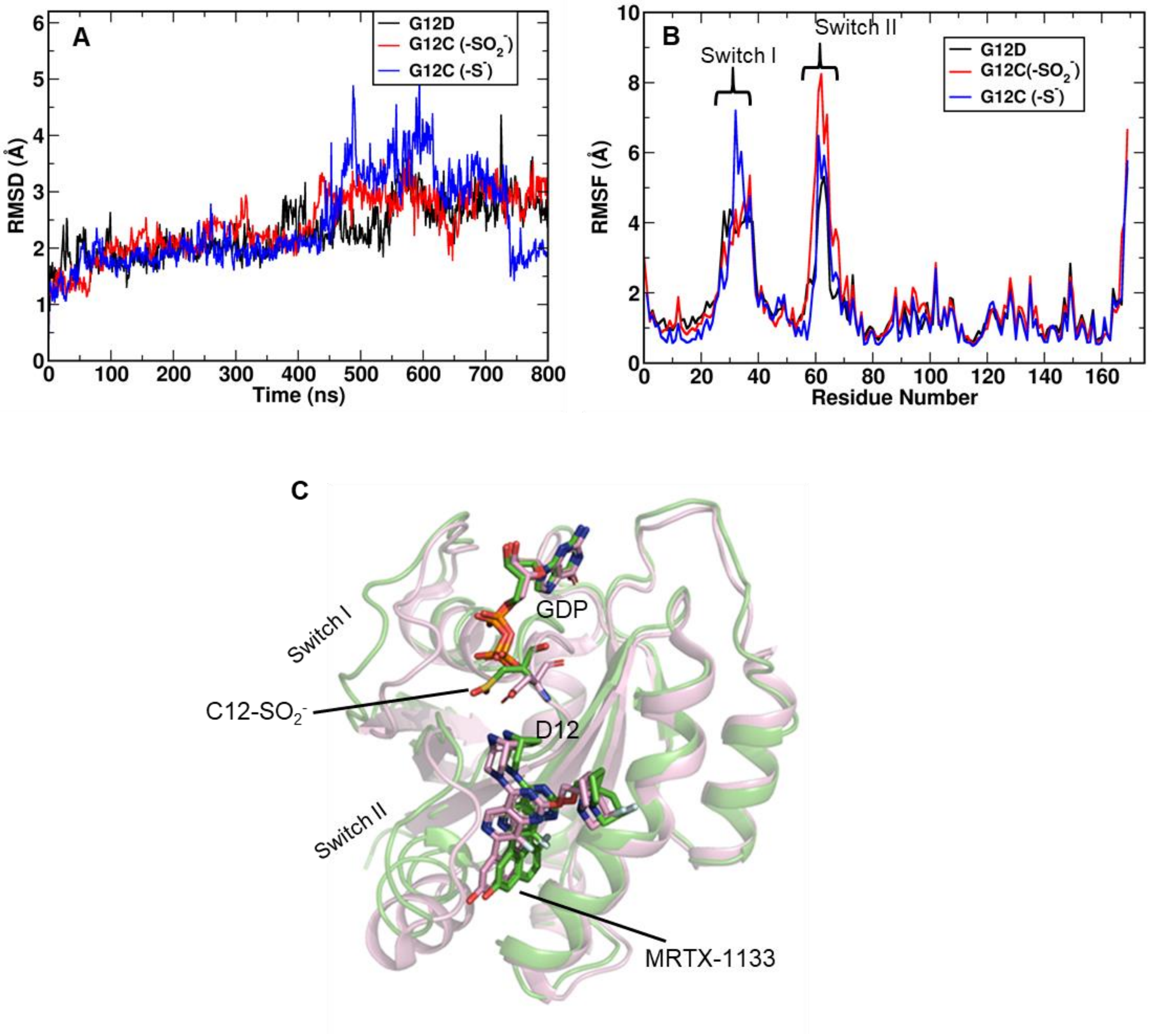

Figure S7. Replicate simulations show similar structural ensembles for KRAS^{G12D} and KRAS^{G12C} (-SO_2^-). Comparison of (**A**) RMSD and (**B**) RMSF plots for GDP-bound KRAS^{G12D}, KRAS^{G12C} (-SO_2^-), and KRAS^{G12C} (-S^-). **(C)** Molecular docking of MRTX-1133 to the representative KRAS^{G12C} (-SO_2^-) conformer (green) shows similar binding to KRAS^{G12D} (pink) (PDB: 7RPZ).

Reference

1. Hansen, R., Peters, U., Babbar, A., Chen, Y., Feng, J., Janes, M. R., Li, L. S., Ren, P., Liu, Y., and Zarrinkar, P. P. (2018) The reactivity-driven biochemical mechanism of covalent KRAS(G12C) inhibitors. *Nature structural & molecular biology* **25**, 454-462
