# Supplementary material for: Oncogenic KRAS G12C: Kinetic and redox characterization of covalent inhibition
Source: J Biol Chem. 2022 Jun 24;298(8):102186. doi: 10.1016/j.jbc.2022.102186 (PMC9352912; doi:10.1016/j.jbc.2022.102186)
Supplement: Supporting information [file mmc2.pdf]

```
[ atomtypes ]
; name at.num  mass   charge  ptype    sigma           epsilon
   SO2   16   32.0660   0.0     A     0.374177        1.966480

 [ CSD ] ; Cysteine sulfinate
 [ atoms ]
  N               NH1            -0.47    0
  HN              H               0.31    1
  CA              CT1             0.07    2
  HA              HB1             0.09    3
  CB              CT2            -0.09    4
  HB1             HA2             0.09    5
  HB2             HA2             0.09    6
  SG              SO2            -0.13    7
  OD1             OC             -0.48    8
  OD2             OC             -0.48    9
  C               C               0.51    10
  O               O              -0.51    11
 [ bonds ]
  CB              CA
  SG              CB
  OD2             SG
  N               HN
  N               CA
  C               CA
  C               +N
  CA              HA
  CB              HB1
  CB              HB2
  O               C
  SG              OD1
 [ impropers ]
  N               -C              CA              HN
```

C	CA	+N	O	
SG	CB	OD2	OD1	

[cmap]

-C	N	CA	C	+N
